# Structural, morphological, and optical properties of TiO_2_ thin films synthesized by the electro phoretic deposition technique

**DOI:** 10.1186/1556-276X-7-357

**Published:** 2012-07-01

**Authors:** Najla Ghrairi, Mongi Bouaicha

**Affiliations:** 1 Laboratoire de Photovoltaique, Centre de Recherches et des Technologies de l’Energie, Technopole de Borj-Cedria, BP 95, Hammam-Lif, Tunis, 2050, Tunisia

**Keywords:** DSC, TiO_2_, Electrophoresis, Physical properties, *I-V* characteristic

## Abstract

In this work, we report the structural, morphological, and optical properties of TiO_2_ thin films synthesized by the electro phoretic deposition technique. The TiO_2_ film was formed on a doped fluorine tin oxide (SnO_2_:F, i.e., FTO) layer and used as a photo electrode in a dye solar cell (DSC). Using spectroscopic ellipsometry measurements in the 200 to 800 nm wavelengths domain, we obtain a thickness of the TiO_2_ film in the range of 70 to 80 nm. Characterizations by X-ray diffraction and atomic force microscopy (AFM) show a polycrystalline film. In addition, AFM investigation shows no cracks in the formed layer. Using an ultraviolet–visible near-infrared spectrophotometer, we found that the transmittance of the TiO_2_ film in the visible domain reaches 75%. From the measured current–voltage or *I-V* characteristic under AM1.5 illumination of the formed DSC, we obtain an open circuit voltage *V*_oc_ = 628 mV and a short circuit current *I*_sc_ = 22.6 μA, where the surface of the formed cell is 3.14 cm^2^.

## Background

**Figure 1:**
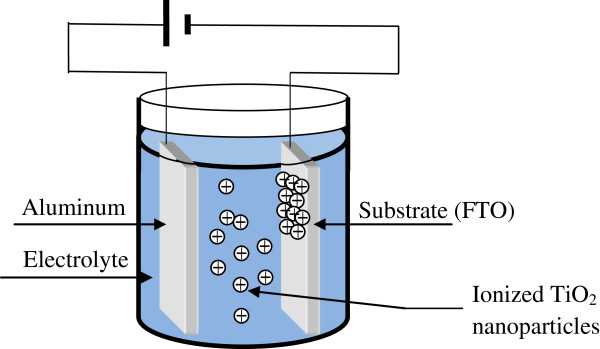
Scheme of the electro phoretic deposition technique.

Dye solar cell (DSC) becomes an interesting photovoltaic generator as thin film solar cells. Despite their low conversion efficiency, they can be very suitable for a big number of applications. In addition to their low cost, DSCs can convert solar radiation into electricity not only with direct (specular) solar lightning, but even with diffused light source. Since the first use of colloidal TiO_2_ nanoparticles by O’Regan and Grätzel in 1991, scientific research in DSCs became promising [1]. Many techniques were used to deposit TiO_2_ film on different substrates, such as liquid phase crystal deposition [2], sol–gel [3-6] (large used), and hydrothermal deposition [7,8]. Abdullah and Sorrell [9] used the electro phoretic deposition (EPD) technique to form TiO_2_ films with different thicknesses on a high purity titanium substrate. Manríquez and Godínez [10] studied the properties of a Ti(III)-doped TiO_2_ film formed by the EPD technique on optically transparent electrode. In this work, we report the structural, morphological and optical properties of TiO_2_ thin films deposited by the EPD technique (Figure 1) on a doped fluorine tin oxide (SnO_2_:F, i.e., FTO) layer. The formed film is used as a photo electrode in the DSC.

The EPD [9-12] is one of the colloid processes in ceramic production. It was discovered since two hundred years ago (1808) and patented in the USA in 1933. This technique was applied in multiple domains; ceramics, coatings, nanoscale assembly, etc [9-12]. It has many advantages such as the homogeneity of the formed film and its fast deposition velocity; it does not require any complex equipment and permits the deposition of thin films on different surface architectures [11] and different textures. The thickness of the film can be controlled via the applied voltage and the deposition duration [11].

In our case, TiO_2_ nanoparticles have a spherical shape in the suspension solution. When deposited by the EPD technique, the non violent arrangement of spherical particles of TiO_2_ on FTO creates void regions (inter spheres); which in turn, gives to the TiO_2_ film its porous structure, with a very high internal surface, which is very useful and may be crucial for the number of dye molecules that we can insert in a DSC and enhance its efficiency.

In this work, the thickness of the formed film is about several tenths of nanometers and was carried out using spectroscopic ellipsometry, as well as the refractive index, *n*, and the extinction coefficient, *k*, in the 200 to 800 nm wavelength range. The structural properties showing a polycrystalline aspect is carried out by X-Ray diffraction (XRD). Morphological investigations using atomic force microscopy (AFM) show no cracks in the film. In addition, the transmittance of the TiO_2_ film in the visible domain reaches 75%. After studying these properties, we fabricate a DSC on which we obtain an open circuit voltage *V*_oc_ = 628 mV and a short circuit current *I*_sc_ = 22.6 μA, where the surface of the cell is 3.14 cm^2^.

## Methods

Thin TiO_2_ films are deposited on FTO substrates using the EPD technique (Figure 1). We used TiO_2_ powder from Aldrich (Sigma-Aldrich Corporation, St. Louis, MO, USA) where 99.7% of the particles have dimensions less than 25 nm. The electrolyte solution is composed of a mixture of 0.02 g of TiO_2_ nanopowder with 30 ml of isopropanol and 10 ml of acetone. Then, we added a solution of iodine (I_2_) dissolved in 5 ml of acetone and 0.5 ml of acetyl-acetone. The electrolyte was dried and ultrasonicated just before deposition. To enhance the TiO_2_ nanoparticles adhesion, the FTO film was first cleaned and exposed to UV-irradiation during 1 h. One of the electrodes is a transparent conductive oxide film, where we used FTO (SnO_2_:F). The latter was deposited on a glass substrate by the pyrolitic technique, and the second electrode is in aluminum (Al) deposited by thermal evaporation. A constant voltage of 80 V was applied between the two electrodes during 5 s. The used voltage and duration are optimized values. Hence, different biases and durations were used by studying the transmittance and the resistivity of the elaborated films. After deposition, the TiO_2_ film was annealed in air at 450 °C during 1 h to enhance the interconnection between nanoparticles. We notice that obtained film has a high adhesion to the FTO substrate. This may be explained by the generated bonding strength between the film and the substrate created during the UV-irradiation of the FTO substrate.

## Results and discussion

### Structural properties

**Figure 2:**
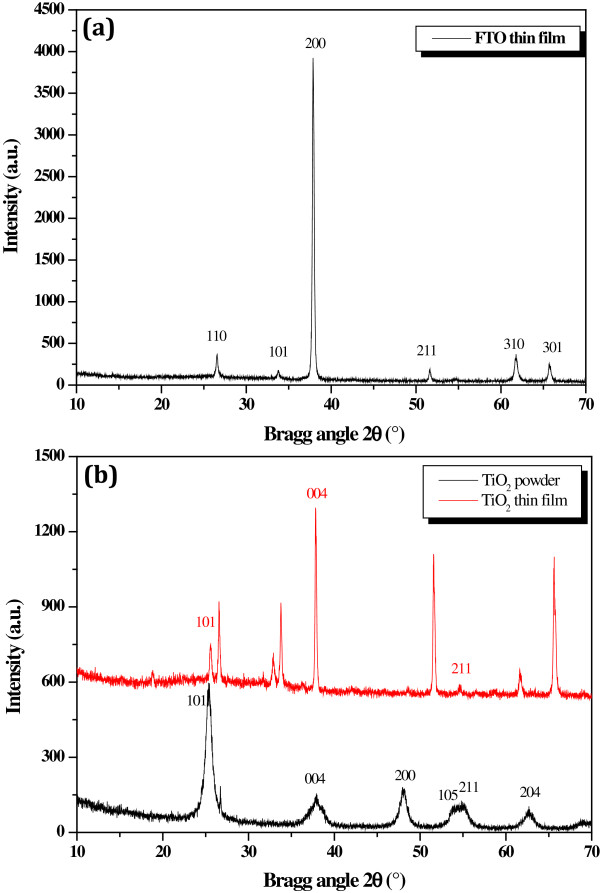
**XRD spectra.(a) FTO film and (b) elaborated TiO**_**2**_** film using TiO**_**2**_**powder.**

We give in Figure 2 three measured XRD spectra. Figure 2a corresponds to the FTO film. Figure 2b is the XRD spectra of TiO_2_ where the black curve corresponds to the TiO_2_ powder and the red curve to the elaborated TiO_2_ film. By taking into account peaks of FTO (Figure 2a) and peaks of the TiO_2_ powder (red curve of Figure 2b), we notice in the XRD spectrum of the formed TiO_2_, three peaks corresponding to the (101), (004), and (211) directions. The peak (004) in both TiO_2_ powder and TiO_2_ film, at the same position of the (200) peak of FTO, was also observed by Radice et al. [12] and Fa-min et al. [13]. Unfortunately, we did not identify the new peak in the fabricated TiO_2_ film that appears near the peak (101) of the FTO layer.

### Morphological properties

**Figure 3:**
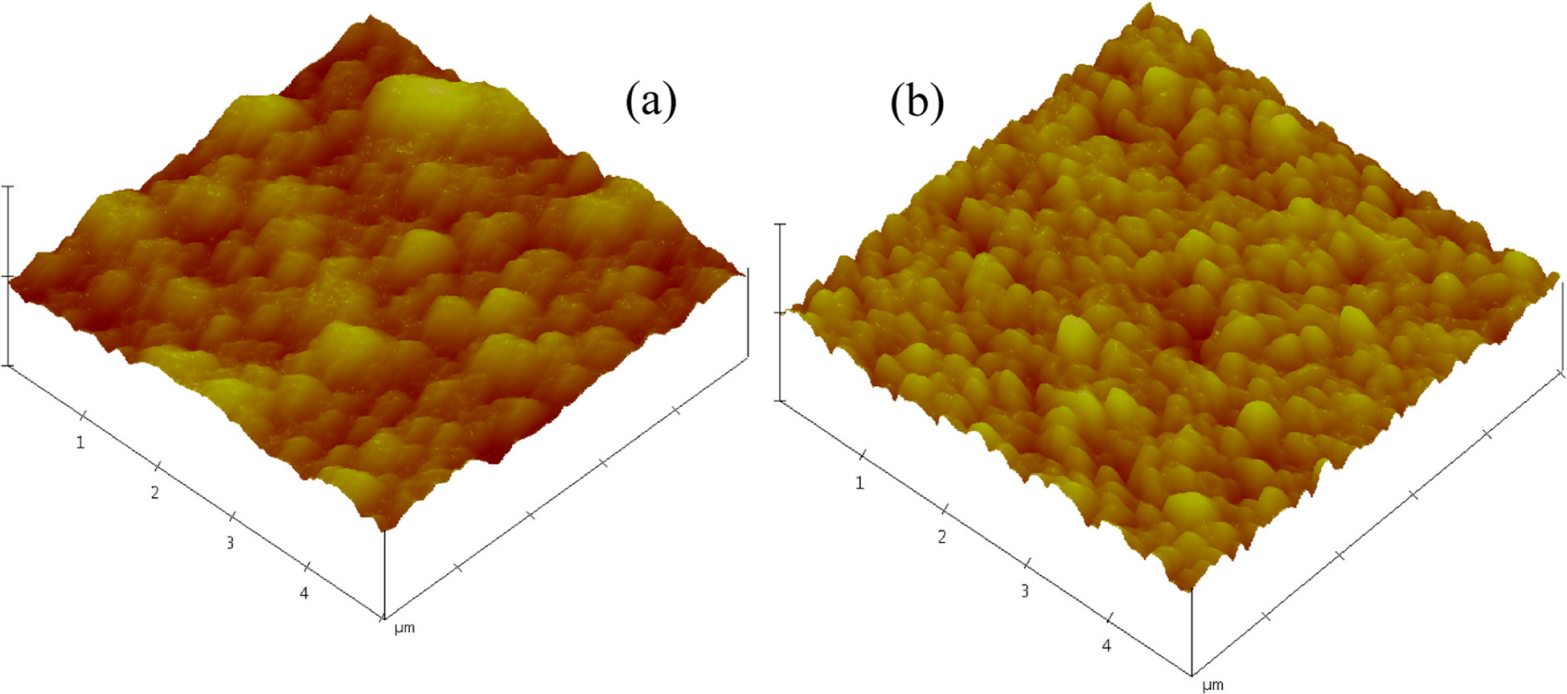
**AFM images.(a) Elaborated TiO**_**2**_** layer and (b) FTO thin film.**

The surface morphology was studied by means of AFM. In Figure 3, we give the AFM image of the TiO_2_ surface (Figure 3a), which was compared to the AFM image of FTO (Figure 3b). The estimated rough surface measurement of the TiO_2_ surface is about 49.8 nm. We can see in the AFM image that the surface reveals different grain sizes of the polycrystalline film, as established by XRD measurements. The sizes of TiO_2_ grains reach the micrometric scale (Figure 3a); however, they have smaller dimensions in the FTO film (Figure 3b). For the imaged region, AFM investigation shows that grain sizes vary from sub micrometric to micrometric dimension. In addition, in regions investigated by AFM, we notice the non compact morphology of the deposited TiO_2_ film and the absence of cracks. This is due to the fact that the suspension solution (Figure 1) contains TiO_2_ nano powder with a spherical shape, and the EPD technique permits their deposition in a porous medium. The latter property and the absence of cracks could be very beneficial for DSCs.

### Ellipsometric study

**Figure 4:**
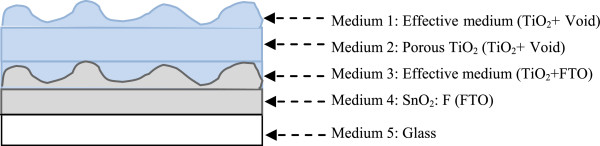
Schematic representation of the structural model of the sample used in the spectroscopic ellipsometer analysis.

**Figure 5:**
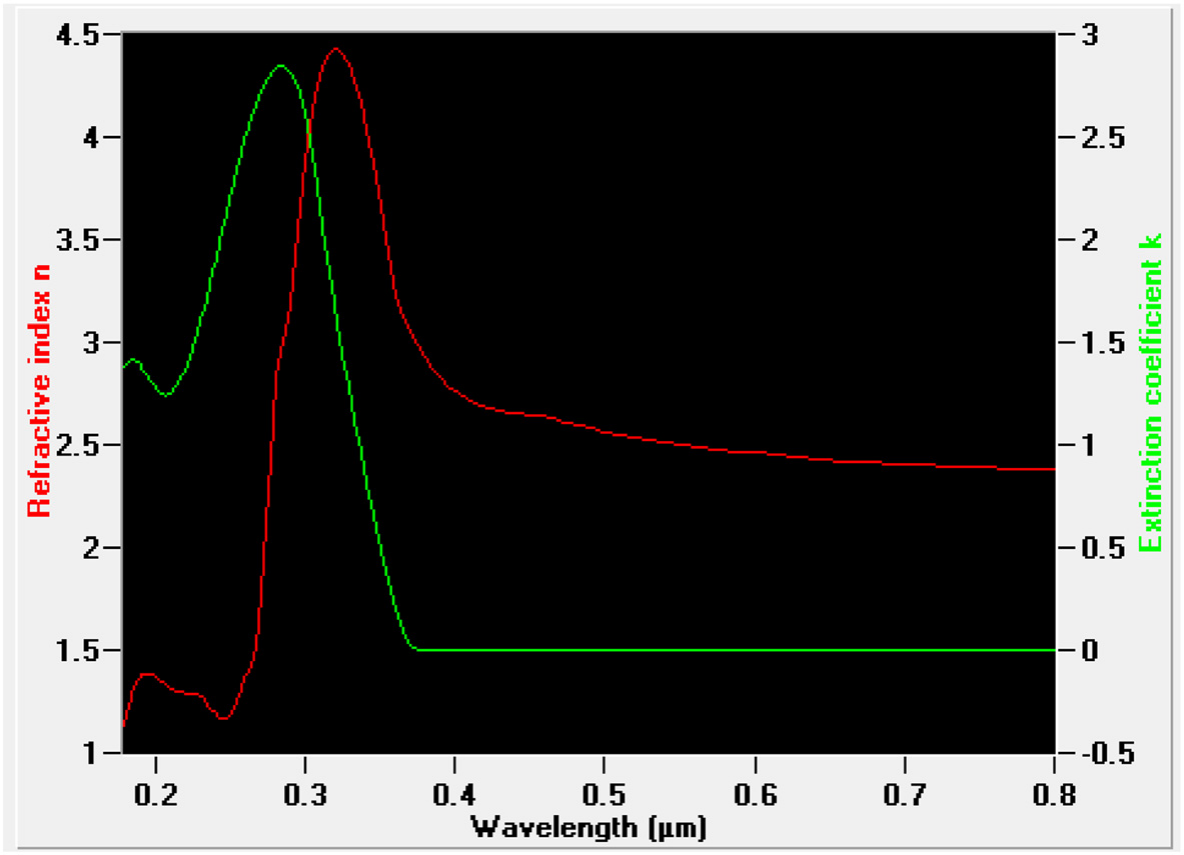
**Refractive index, *n* , and extinction coefficient, *k* , of the TiO**_**2**_**film.**

To carry out the refractive index and the extinction coefficient, we use an ultraviolet–visible near-infrared (UV–vis-NIR) GES5 spectroscopic ellipsometer from Sopra (Uttar Pradesh, India). Measurements were achieved with an average value of incident angle about 60° in a wavelength range of 200 to 800 nm at ambient temperature. This choice of angle is justified by the best minimization of the noise level. After collecting all measurements, the determination of the physical parameters is based on the method of how to choose the best model that enables good fitting results of theoretical curves of cos(Δ) and tan(Ψ) to experimental ones as a function of the wavelength. In Figure 4, we give a schematic representation of the sample as considered in the theory to obtain the best fit. The sample is considered as formed by five mediums. In order to enhance the fitting results, all layers are supposedly homogenous, transparent, and isotropic to use the Bruggeman effective medium theory (EMA). As shown in Figure 4, we used following three basic mediums: medium 2 (porous TiO_2_: TiO_2_ + void), medium 4 (FTO), and medium 5 (glass). At the tow interfaces, we considered effective mediums formed by two phases of each one. Hence, between air and porous TiO_2_, we considered medium 1. Between medium 2 and medium 4, we consider the effective medium 3. These considerations are due to the surface roughness as obtained by AFM investigations. Utilizing the model structure given in Figure 4, we represent in Figure 5 the refractive index, *n*, and the extinction coefficient, *k*, as a function of the incident wavelength. The obtained values of *n* and *k* are very close to those in the literature [14]. In Table 1, we give the values of the films’ thicknesses obtained after fitting using the EMA theory.

**Table 1:** **Obtained thicknesses of mediums considered in Figure**4

**Medium**	**Thickness (nm)**
Medium 1: TiO_2_/void	68.70
Medium 2: porous TiO_2_	4.36
Medium 3: SnO_2_/TiO_2_	5.05
Medium 4: SnO_2_:F	1,021.60
Medium 5: glass	-

### Transmittance

**Figure 6:**
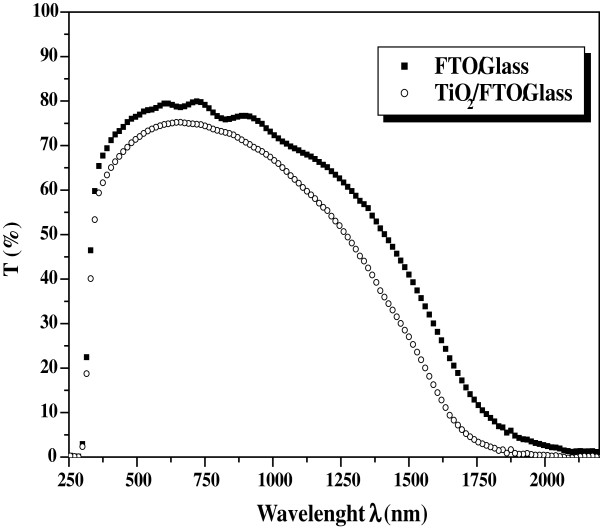
**Measured transmittance spectra of FTO/glass and TiO**_**2**_**/FTO/glass films vs. wavelength.**

Transmittance (*T*) of the film was carried out using a UV–vis-NIR spectrophotometer in the wavelength range from 250 to 2,500 nm. The transmittance of the TiO_2_ film is plotted in Figure 6 as well as the transmittance of the FTO film which is used for comparison. We notice a very useful transparency of the film in a large wavelength domain from 300 to 1,100 nm. Hence, the values of *T* reach 75% in the visible domain, which is very functional for DSC applications, since we will use it as the photo electrode. In addition, one can notice that as compared to the FTO/glass film, the transmittance is not diminished severely when we form the TiO_2_ film (Figure 6).

From the measured transmittance *T* given in Figure 6, we deduce the absorption coefficient *α* using the following relation [15]:

(1) $$\alpha \approx \frac{1}{d}\ln\left(\frac{1}{T}\right)$$

where *d* is the thickness of the film (Table 1) and *T* is its transmittance.

It is established that TiO_2_ has direct and indirect band gaps [6]. To determine values of these forbidden energies, we use the expression in Equation 2.

**Figure 7:**
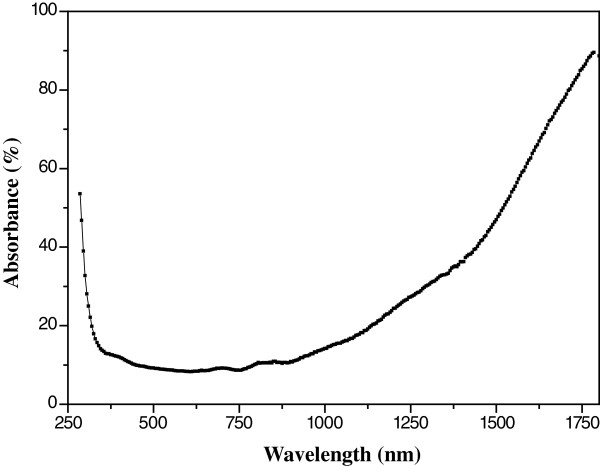
Absorption coefficient α as a function of the incident wavelength.

**Figure 8:**
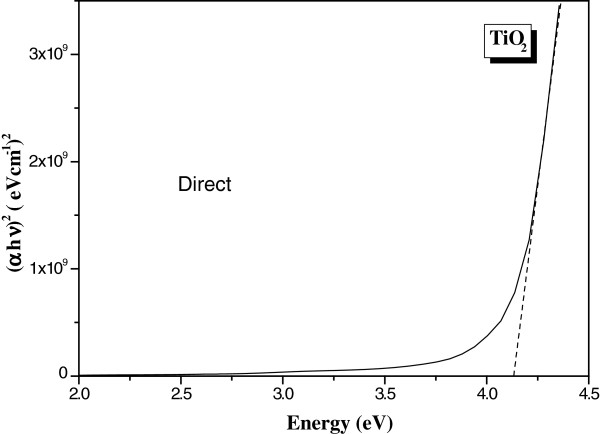
**Plot of (αhν)**^**2**^**vs. (hν) for the estimation of the direct gap energy value.**

**Figure 9:**
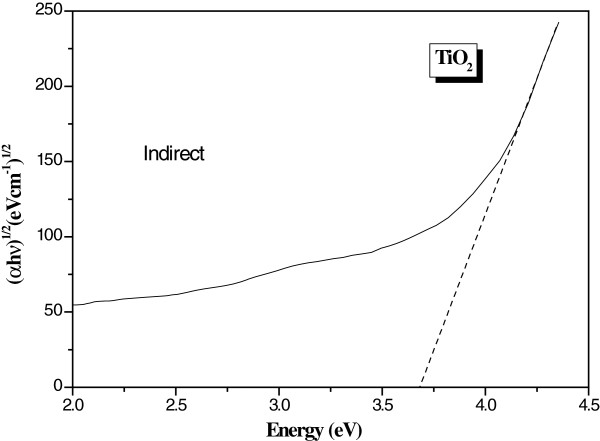
**Plot of (αhν)**^**1/2**^**vs. (hν) for the estimation of the indirect gap energy value.**

The relationship between the absorption coefficient *α* and the incident photon energy is given by the relation (Equation 2) as follows [16]:

(2) $$\alpha hv=A{\left(hv-E_{g}\right)}^{m}$$

where A is a constant depending on the transition probability and *m* is equal to ½ for indirect gap and 2 for direct gap. The usual method to calculate the band gap energies is to plot ${\left(\alpha hv\right)}^{\frac{1}{m}}$ as a function of the incident radiation energy (hν) [17]. The absorption coefficient α as a function of the incident wavelength is plot in Figure 7. The band gap values are determined by extrapolating values of the absorption coefficient α to zero. Figures 8 and 9 are the plot of ${\left(\alpha hv\right)}^{\frac{1}{m}}$ vs. incident energy (hν). As represented in these figures, we found 4.14 and 3.40 eV for the direct and indirect band gap energies, respectively. These values are very close to those obtained by Janitabar et al. [6] for a TiO_2_ film elaborated by the sol–gel templating technique. The obtained value of the band gaps, especially for direct transition, could be due to the radius of quantum-sized particles [6].

### Current–voltage characterization

**Figure 10:**
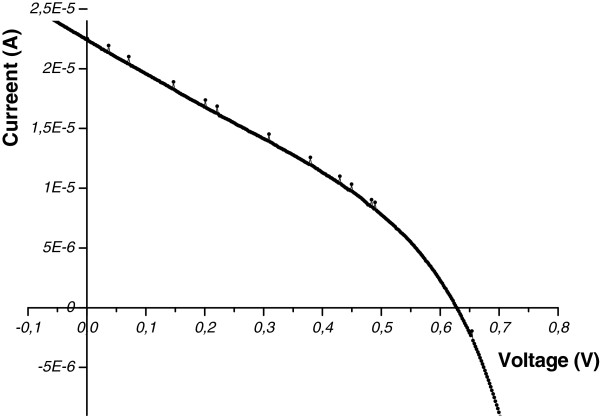
AM1.5 current–voltage characteristic of the formed DSC.

After depositing and analyzing the TiO_2_ film, the photo electrode was immersed for 24 h in N3 dye solution. For the fabrication of the DSC, we used KI/I_2_ electrolyte dissolved in acetonitrile as solvent. The counter electrode is in aluminum on FTO. The surface area is 3.14 cm^2^. In Figure 10 we give the measured current–voltage (*I-V*) characteristic of the formed DSC performed at AM1.5 illumination. We obtain an open circuit voltage *V*_oc_ = 628 mV and a short circuit current *I*_sc_ = 22.6 μA. However, we notice that due to the fact that we used aluminum in the counter electrode instead of platinum, we observed a relatively rapid degradation on the counter electrode of the DSC as a function of time. This degradation could be at the origin of the leakage current, leading to a decrease of the shunt resistance of the cell as observed in the *I-V* curve for low voltage.

## Conclusions

In this paper we give the structural, morphological, and optical properties of TiO_2_ films which are formed using the EPD technique. By analyzing the elaborated film by means of XRD, AFM, ellipsometry, and UV–vis-NIR spectrophotometry, we established that EPD technique permits the formation of a film that could be useful for DSC application. Hence, the XRD characterization shows polycrystalline films. The AFM investigations show no cracks in the film. The spectroscopic ellipsometry analysis permits us to deduce the thickness of the TiO_2_ film which we found in the range of 70 to 80 nm, as well as the refractive index, *n*, and the extinction coefficient, *k*, in the 200 to 800 nm wavelength range. In addition, we found that the transmittance of the TiO_2_ film in the visible domain reaches 75% in a large spectral range.

From the *I-V* characteristic measured at AM1.5 of the formed DSC, we obtain *V*_oc_ = 628 mV and *I*_sc_ = 22.6 μA. Though we remark that due to the fact that we used aluminum in the counter electrode instead of platinum, we observed the degradation on the counter electrode of the DSC. This degradation might create a leakage current, leading to a decrease of the shunt resistance of the cell, as it can be observed in the *I-V* curve for low voltage.

## Competing interests

The authors declare that they have no competing interests.

## Authors’ contributions

NG prepared samples, performed all characterizations and participate in writing the paper. MB supervised the work, helps in the interpretations, and wrote the text. Both authors read and approved the final manuscript.
